# Automatic detection of pathological myopia using machine learning

**DOI:** 10.1038/s41598-021-95205-1

**Published:** 2021-08-16

**Authors:** Namra Rauf, Syed Omer Gilani, Asim Waris

**Affiliations:** Department of Biomedical Engineering and Sciences, National University of Sciences and Technology (NUST), Islamabad, 44000 Pakistan

**Keywords:** Health care, Biomedical engineering

## Abstract

Pathological myopia is a severe case of myopia, i.e., nearsightedness. Pathological myopia is also known as degenerative myopia because it ultimately leads to blindness. In pathological myopia, certain myopia-specific pathologies occur at the eye’s posterior i.e., Foster-Fuchs’s spot, Cystoid degeneration, Liquefaction, Macular degeneration, Vitreous opacities, Weiss’s reflex, Posterior staphyloma, etc. This research is aimed at developing a machine learning (ML) approach for the automatic detection of pathological myopia based on fundus images. A deep learning technique of convolutional neural network (CNN) is employed for this purpose. A CNN model is developed in Spyder. The fundus images are first preprocessed. The preprocessed images are then fed to the designed CNN model. The CNN model automatically extracts the features from the input images and classifies the images i.e., normal image or pathological myopia. The best performing CNN model achieved an AUC score of 0.9845. The best validation loss obtained is 0.1457. The results show that the model can be successfully employed to detect pathological myopia from the fundus images.

## Introduction

Pathological myopia (PM) is a severe case of myopia or nearsightedness. It is also called degenerative myopia due to myopia-specific pathology at the posterior, i.e. Foster-Fuchs’s spot, Cystoid degeneration, Liquefaction, Macular degeneration, Vitreous opacities, Weiss’s reflex, Posterior staphyloma, etc. PM is caused by the biomechanical forces linked to the eye’s axial elongation. These forces cause the stretching of the ocular layers and progressive thinning of the retina, sclera, and choroid^1^. It may ultimately lead to blindness because it’s progressive and irreversible.

Wong et al*.* study predict that 50% and 10% of the world population will have myopia and high myopia by 2050. The higher cases of myopia will have the potential to result in vision impairment due to myopic macular degeneration or its comorbidities. Myopia would become the leading cause of permanent blindness by 2050^2^.

The goal of this study is to contribute towards developing an automatic, accurate, and non-invasive diagnostic method for PM patients. Clinical diagnosis of pathological myopia depends upon the examination of the fundus images. The decision taken by a doctor of PM depends upon various ophthalmic examinations besides the funduscopic exam. So the study focuses on designing a CNN model for the stated cause. With promising results, the study can be extended to a clinical setup in the future. Thus, the time and resources of both the doctor and the patient could be saved. Because the condition will be diagnosed automatically solely based on the fundus images of a patient.

Some related work includes that of Mengdi Xu et al*.* which focused on using color moments, local binary patterns (LBP), and histograms of oriented gradients (HOG) feature for the automated detection of the tessellated fundus. Bag-of-words (BOW) is used in extracting HOG and LBP features. Support Vector Machine (SVM) is used for classification^3^. Anindita Septiarini et al*.* proposed a five-step method for peripapillary atrophy detection in retinal fundus images. Mean, standard deviation, smoothness, third moment, uniformity, and entropy are chosen as characteristic features. Back Propagation Neural Network (BPNN) is used for classification^4^. Zhou Zhang et al*.* study focuses on identifying a compact feature set that can be used for predicting pathological myopia. The study proposes a minimum Redundancy- Maximum Relevance (mRMR) based classification approach. The features set are composed of the information extracted from the fundus images and the screening exam. It is concluded that the accuracy obtained using mRMR classifiers is approximately higher than simply using a support vector machine^5^. Jiang Liu et al*.* focused on learning the most relevant visual features of pathological myopia by using machine learning and computer vision techniques. Scale-invariant feature transform (SIFT) feature extraction of the green channel is done. K-mean clustering is employed to generate a codebook after extraction of all the SIFT features from training images^6^. Xiangyu et al*.* developed a deep learning (DL) architecture with a convolutional neural network for automatic detection of glaucoma. Region of interest (ROI) image is given as input to the deep Convolutional Neural Network (CNN)^7^. Jiang Liu et al*.* worked on the detection of pathological myopia based on the presence of peripapillary atrophy (PPA). Features are generated from the combination of individual and relativistic metrics. These texture-based features are given to an SVM classifier^8^. Yanwu Xu proposed a Multiple Kernel Learning (MKL) technique for the detection of ocular diseases including pathological myopia. The study compares the MKL^*clm*^ with two baseline methods i.e. standard SVM and MKL method under three sets of experiments^9^.

## Method and experimental setup

Deep learning emerged as a subfield of machine learning. It enables the computer to make complex concepts from simpler concepts^10^. The ‘deep’ in deep learning refers to the multiple layers of representations^11^. A simple deep learning model is composed of one input layer, one output layer, and a hidden layer (can be multiples).

### Overview of deep learning architecture

Convolutional neural networks, a deep learning model, are also known as “convnets.” It takes its name from the mathematical operation of “convolution” that it employs. CNN is mostly used in the field of computer vision particularly in image classification problems. The key terms in the architecture of a CNN (designed in this research) are described below:

### Convolutional layer

In terms of image processing, convolution is nothing else than a simple element-wise matric multiplication and sum of a filter and an input image. It helps to learn the local patterns^11^. Patch s, depth of output feature map (number of filters used in the convolution), and stride are parameters of the convolutional layer. The convolutional operation:

$$s(t)=(x*w)(t)$$

x is the first argument to the convolution and is called input. If the input to the CNNs is an image, then this x constitutes the multidimensional array of pixel values. w is the second argument is called the kernel. It is the filter with which the multiplication is done. s(t) is the output of the operation. It is also called a feature map.

### Pooling layer

The pooling layer receives the output of the convolutional layer and extracts features from each activation map. There are two main types of pooling: Max pooling and Average pooling. Max pooling is focused on this work. Because it’s more informative to observe the maximal presence of the features than their average presence^11^. After each max polling operation, the feature map’s size gets halved.

### Fully connected layer

The neurons of the fully connected layer receive the input from every neuron in the pooling layer. These incoming inputs are flattened out i.e. vectorization. The process of classification starts at this layer^11^. A fully connected layer’s output is equal to the number of classes in the classification task^12^.

### Activation function

The activation function is non-linear. It regulates the firing of a neuron i.e. whether the incoming information is enough to activate the neuron.

### Batch normalization

Normalization makes the various samples observed by a machine-learning model less different from each other, helping the model to learn and generalize better to new data^11^. It is usually employed after a fully connected/convolutional layer and non-linearity. It should be noted that during the process, we are not changing the weights but only normalizing the inputs to each layer. The equation shows how it’s computed.

$${\widehat{x}}^{\left(k\right)}=\frac{{x}^{\left(k\right)} - E\left[{x}^{\left(k\right)}\right]}{\sqrt{Var\left[{x}^{\left(k\right)}\right]}}.$$

### Optimizer

The job of an optimizer is to determine how a network will get updated based on a loss function. A feedback signal is used to adjust the value of the weight in the direction of a lower loss score. It implements it by the Backpropagation algorithm. Backpropagation starts with the final value of the loss and works backward from the top to the bottom layers, using the chain rule to measure the contribution of each parameter in the loss value^11^.

### Loss function

It’s also called the Cost function. It’s the quantity that is tried to be minimized during the training process^11^. All the networks focus on reducing the loss.

### Dropout

It is one of the most common and effective regularization techniques. It randomly drops out a number of the layer features during training. The dropout rate determines the fraction of the features that’ll be zeroed out. Its common settings are between 0.2 and 0.5. It helps reduce overfitting^11^. Overfitting refers to the situation where the network has good performance on the training data but fails to generalize.

### Weight regularization

It’s employed to minimize overfitting of the model. Overfitting is reduced by putting constraints on the network’s complexity. It is achieved by adding a cost linked with having large weights to the loss function. The cost is of two types; L1 regularization and L2 regularization. Focus is on the L2 regularization also known as weight decay. In it, the added cost is proportional to the square of the value of the weight coefficients^11^.

### Mini-batch

The whole training data is divided into small equal samples called mini-batch. Thus mini-batch is a subset of the training data. It is specified by ‘batch size.’ It reduces the computation cost and saves memory.

## Experimental setup

The experimental setup consists of three main steps. These are discussed below:

### Preprocessing phase

#### (a) Grayscale conversion

**Figure 1:**
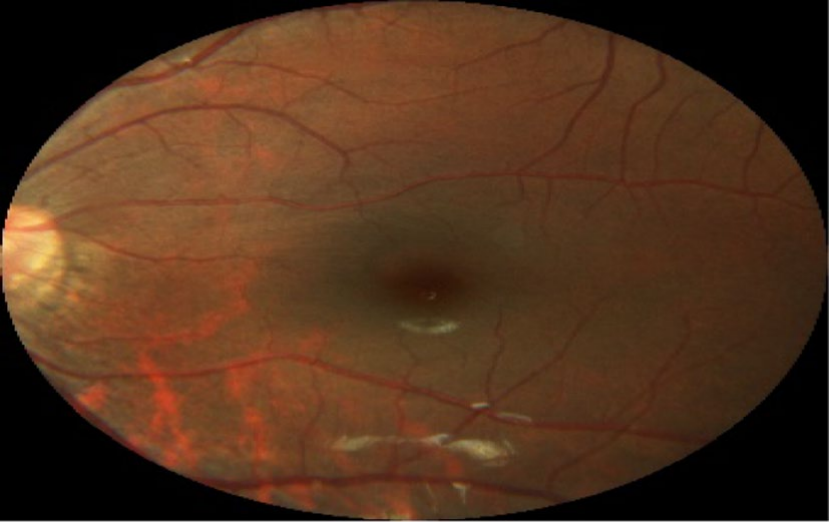
Original image (1444 × 1444 × 3)^14^.

**Figure 2:**
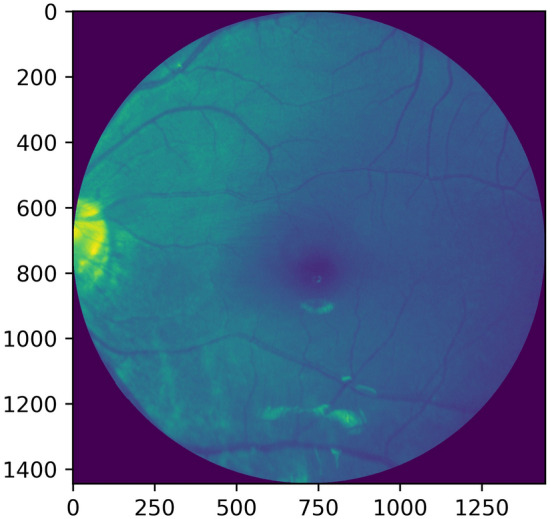
Grayscale image (1444 × 1444).

The original input images are color images, i.e. RGB format. Figure 1 shows the original image having three channels i.e. red, blue, and green. These input images are preprocessed and converted into grayscale. Figure 2 shows the grayscale image.

#### (b) Resize

**Figure 3:**
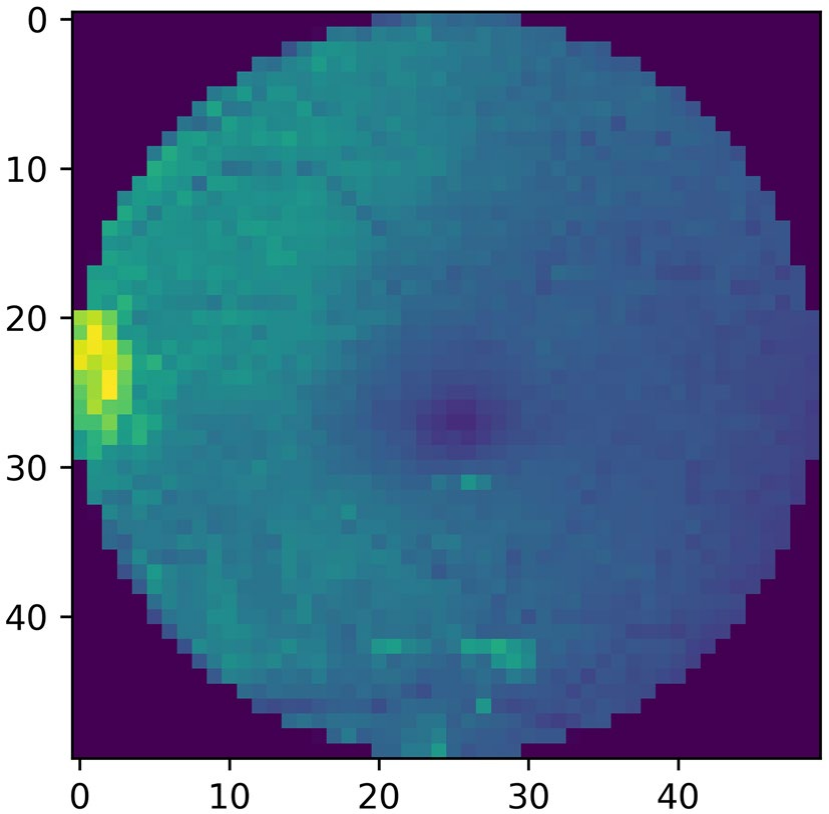
Image size (50 × 50).

The original image size is very large i.e. 1444 × 1444 × 3. Resizing is necessary for reducing the computational load, train the network faster, and reduce the time required by training. However, it is necessary that the resizing should not be done to a value that’ll lose the useful information. Figure 3 shows a grayscale fundus image that is resized.

#### (c) Shuffle

The input images are shuffled in a preprocessing step. It’s necessary as it removes biases from the process by sorting the images randomly. Also, network performance is increased. Because if images are not shuffled, the network gets to train on only a specific category (i.e. Normal) and its performance degrades for another category (i.e. PM).

#### (d) Normalization

It’s achieved by dividing each pixel value by the maximum value of a pixel i.e. 255. This bounds the pixel intensity values to be in between 0 to 1.

#### (e) Histogram equalization

**Figure 4:**
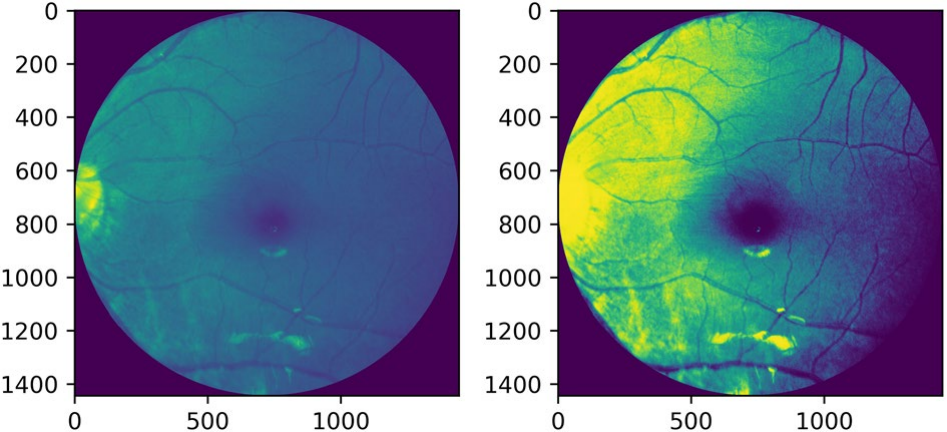
Grayscale image and its histogram equalization.

**Figure 5:**
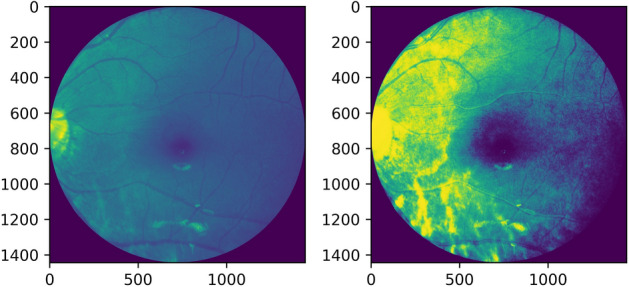
Red channel extracted image and its histogram equalization.

Histogram equalization or linearization is applied so that a high contrast image could be produced by increasing the range of intensity values. Figures 4, 5 shows the operation done on both, i.e. a grayscale image and a red channel image.

**Figure 6:**
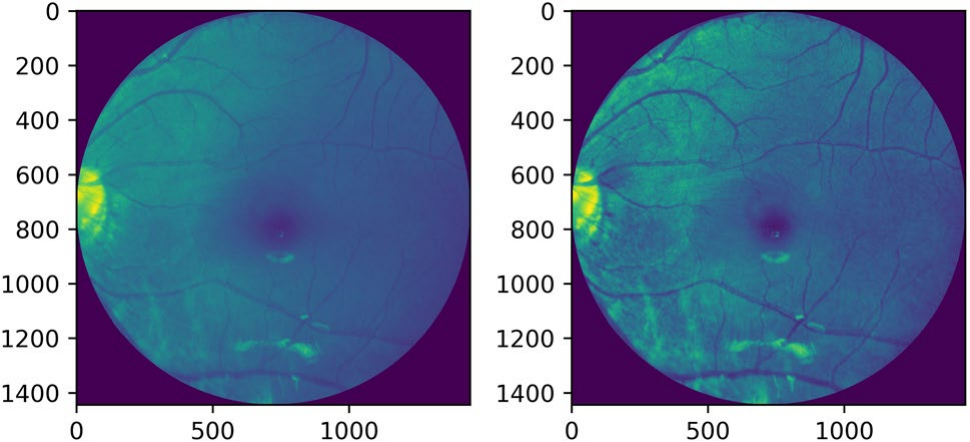
Grayscale image and its adaptive histogram equalization.

**Figure 7:**
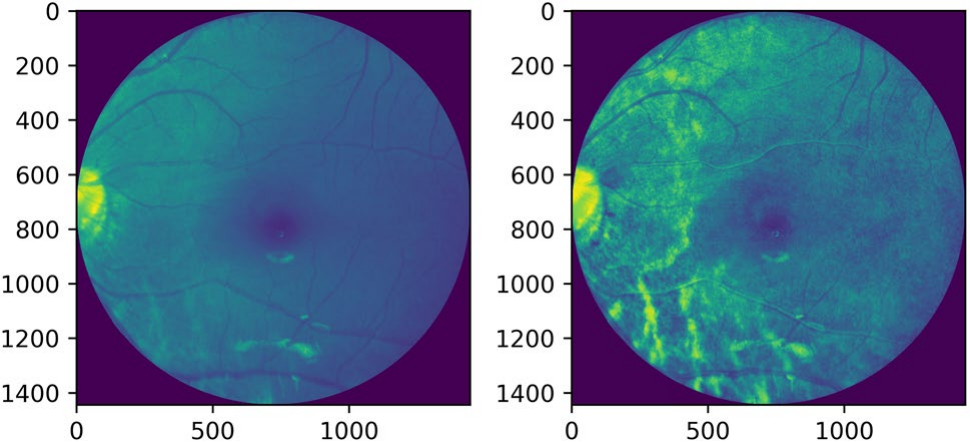
Red channel extracted image and its adaptive histogram equalization.

Adaptive histogram equalization is also employed as seen in Figures 6, 7. The image gets divided into small blocks known as ‘tiles’ (tile size is 8 × 8 in OpenCV by default). To avoid noise amplification, contrast limiting is done. If any histogram bin is above the specified contrast limit (40 by default), these pixels are clipped out and uniformly distributed to other bins before the application of histogram equalization^13^.

#### (f) Red channel extraction

**Figure 8:**
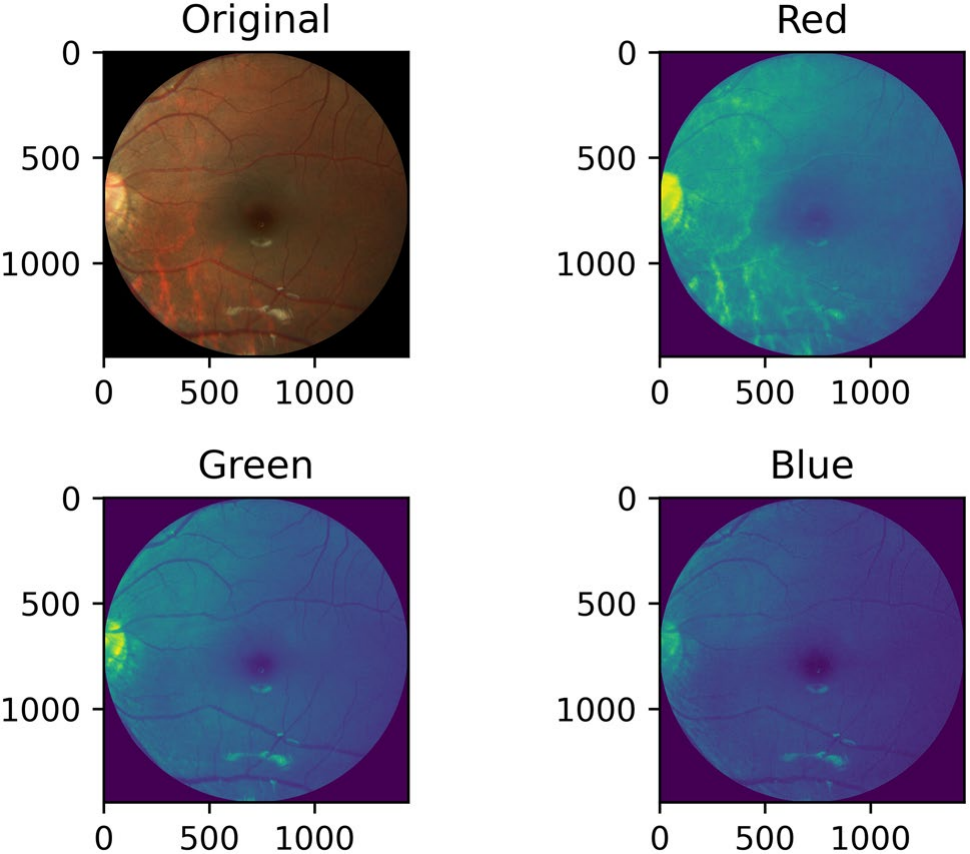
Original image and the three extracted channel images, i.e. red, green, and blue.

**Figure 9:**
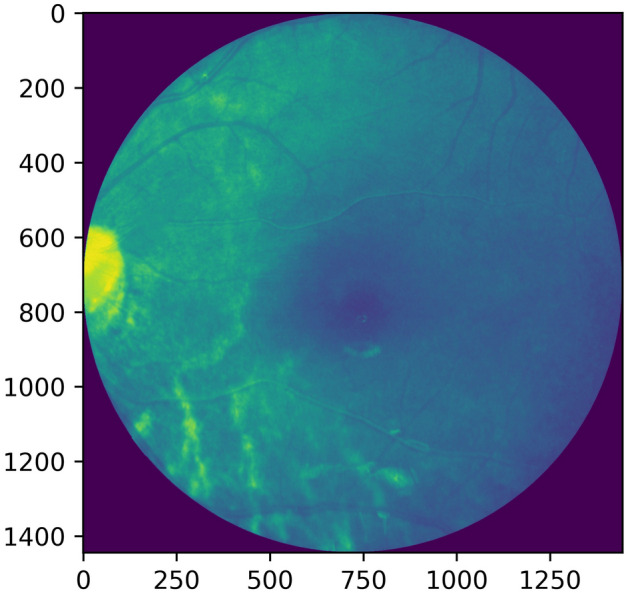
Red channel extracted fundus image.

Visual observation of the fundus images reveals a high content of red color. Based on this observation, the red channel from the original image is extracted and preprocessed. Figure 8 shows the original image with the three channels. Figure 9 shows the extracted red channel.

### Training phase

**Figure 10:**
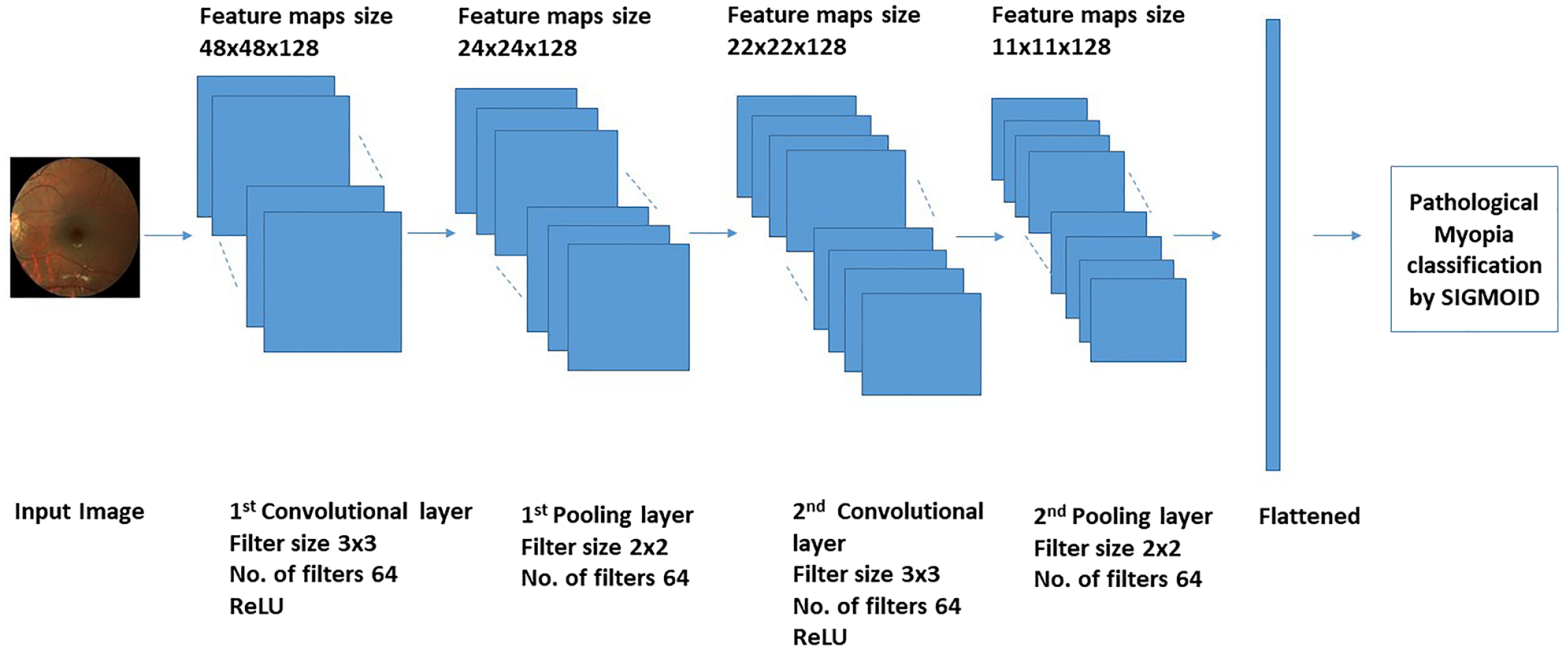
The best performing CNN model i.e. 2C-128N-0D.

Figure 10 shows the best performing CNN model architecture. In the second step, the CNN model is trained on the given pre-processed training input data. The model is evaluated based on the validation set. It helps us in adjusting hyperparameters by giving us information regarding network performance. To find the best performing model, hyperparameter optimization is done on TensorBoard with a validation dataset.

### Testing phase

In this last step, the CNN model that had been trained is now tested on the test images. The labels of the test images are not available. The prediction values for the test images are calculated. These prediction values are probability values showing the risk of PM. The higher the probability value the greater is the risk of PM. These prediction values are checked on the Baidu platform that calculates Area under the curve score (AUC). Higher values of AUC means good result.

### Dataset

PALM challenge 2019 dataset is used in this study. PALM is a satellite event hosted by the International Symposium on Biomedical Imaging (ISBI-2019) in Italy. The dataset contains a total of 400 training images for which labels (normal 161 & myopic 239) are provided and 400 test images for which the labels are not provided. Both these sets contain images of a normal eye and a pathological myopic eye. The size of each fundus image is 1444 × 1444 × 3 (RGB image). During the training of CNN, we have not explicitly defined a validation set; rather, we have used the ‘*validation_split* = *0.1*’ command in Keras to separate 10% of the training data into a validation set. The database can be found at https://palm.grand-challenge.org/^14^.

### Ethical approval

This article does not contain any studies with human participants or animals performed by any of the authors.

## Results and analysis

The CNN algorithm is developed in Spyder and is executed on Intel Core i7 Quad-Core CPU (8 GB RAM) at 2.60 GHz with NVIDIA GPU (4 GB). The accuracy, loss, and AUC score are the evaluation metrics. The CNN model is tested for different optimizers. Among which the best performing optimizer is selected i.e. ADAM. Various preprocessing techniques are checked. Dropout of 0.3 and L2 regularization of 0.001 is added to the best performing network (Table 1) to reduce overfitting.

**Table 1:** Different preprocessing steps and their respective AUC score.

Preprocessing step	AUC score
Grayscale image, resize, shuffle, normalization	0.9593
Grayscale, resize, shuffle, normalization, batch normalization	0.9745
Red channel image, resize, shuffle, normalization	0.9829
Red channel image, resize, shuffle, normalization, batch normalization	**0.9845**
Grayscale image, histogram equalization, resize, shuffle, normalization, batch normalization	0.9723
Grayscale image, adaptive histogram equalization (CLAHE), resize, shuffle, normalization, batch normalization	0.9761
Red channel image, adaptive histogram equalization (CLAHE), resize, shuffle, normalization, batch normalization	0.9707

Bold shows the best combination for preprocessing step.

**Figure 11:**
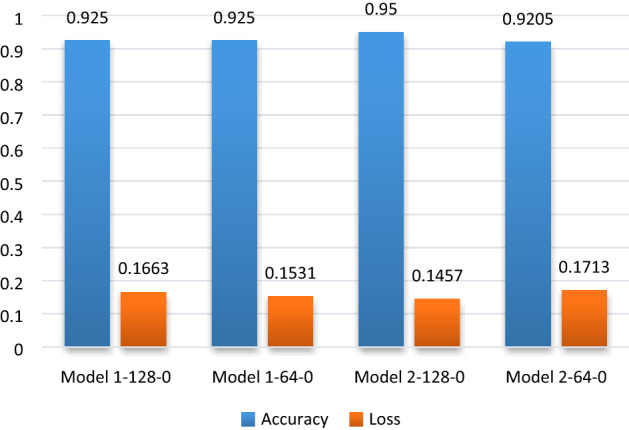
Performance of the models based on evaluation metrics.

Tensor board is used to optimize the designed model having the best AUC score. It is achieved by checking different model capacities, i.e. different number convolutional layer (1, 2, 3), layer size (32, 64,128), and dense layer (0, 1, 2; these are added before the last dense layer of each model). There is a total of 27 different models. Figure 11 shows the four selected models out of 27, selected based on their better performance.

Our proposed method is compared with other state of the art methods in Table 2.

**Table 2:** Comparison analysis.

Author	Accuracy	AUC
Mengdi Xu et al	98%	–
Anindita Septiarini et al	96%	–
Jiang Liu et al	90.6 + − 1.0%	0.964 + −0.007
Xiangyu et al	–	0.887
Jiang Liu et al	87.5%	–
Yanwu Xu et al	–	0.946 + − 0.010
Proposed method	95%	0.9845

## Discussion and conclusion

A convolutional neural network designed for automatic detection of pathological myopia is the aim of this research project. A simple CNN model is designed using the Spyder platform (Python 3.7). A series of tests are conducted on the designed CNN model. All these tests are focused on obtaining the best performing network. From best performing CNN, we mean the CNN model that has the least validation loss. In an attempt to do so, several preprocessing steps are added to the tests. These preprocessing steps include resizing of the image, conversion to a grayscale image, and extraction of red channel image, shuffling of the images, histogram equalization, adaptive histogram equalization, and normalization. Different optimizers and activation functions are also tested.

Furthermore, batch normalization is added and different learning rates (default learning rate performed better) are checked. The results of each test are being evaluated based on the AUC score i.e. area under the curve. However, the best AUC score does not ensure a perfect model i.e. free of overfitting. To overcome the overfitting problem, dropout and L2 regularization are added to the network architecture. The tensor board is used for network optimization. It’s achieved by analyzing the graphs for different model capacities.

In conclusion, the preprocessing step of red channel extraction and the batch normalization gives the best AUC score. Comparatively, neither the histogram equalization nor the adaptive histogram equalization has any contribution towards improving the AUC score. Adam optimizer, ReLU, and sigmoid activation functions showed the best performance for the selected CNN model. The overfitting is significantly removed by adding dropout and L2 regularization. The best performing CNN model is 2C-128N-0D i.e. two convolutional layers, 128 layer size, and no multiple dense layers. Figure 10 shows the architecture of this model. The proposed classification model is trained from scratch and is not dependent on transfer learning like Cefas Rodrigues Freire et al.^15^ and does not use data augmentation like Jaydeep Devda et al.^16^. The validation loss of this CNN model is 0.1457. The results of the proposed model are compared to another state of the art techniques in Table 2. The comparison shows that our proposed CNN model performs better than the rest, based on the Accuracy and AUC values.

The prime focus of this research is to work on devising an efficient and accurate diagnostic method for pathological myopia detection. This study could be extended to the diagnostic study of other types of eye-related complications i.e. glaucoma, retinopathy, etc. Furthermore, it can be used in deep learning research to analyze the efficiency of this technique and several others for this condition.

## Abbreviations

PM: Pathological myopia
ANN: Artificial neural network
CNN: Convolutional neural network
RGB: Red, green blue
Acc: Accuracy
AUC: Area under the curve
